# Expenditure of biological drugs for rheumatoid arthritis treatment in the Brazilian public health system

**DOI:** 10.11606/s1518-8787.2023057004280

**Published:** 2023-07-18

**Authors:** Tacila Pires Mega, Rondineli Mendes da Silva

**Affiliations:** I Fundação Oswaldo Cruz Escola Nacional de Saúde Pública Sergio Arouca Programa de Pós-Graduação em Saúde Pública Rio de Janeiro RJ Brasil Fundação Oswaldo Cruz. Escola Nacional de Saúde Pública Sergio Arouca. Programa de Pós-Graduação em Saúde Pública. Rio de Janeiro, RJ, Brasil; II Fundação Oswaldo Cruz Escola Nacional de Saúde Pública Sergio Arouca Departamento de Política de Medicamentos e Assistência Farmacêutica Rio de Janeiro RJ Brasil Fundação Oswaldo Cruz. Escola Nacional de Saúde Pública Sergio Arouca. Departamento de Política de Medicamentos e Assistência Farmacêutica. Rio de Janeiro, RJ, Brasil

**Keywords:** Arthritis, Rheumatoid, Delivery of Health Care, Public Expenditures, Biological Products, Pharmaceutical Services

## Abstract

**OBJECTIVE:**

This work aims to analyze the quantity and expenses related to biological drugs used for the treatment of rheumatoid arthritis (RA) in outpatient public care within the Brazilian Unified Health System (SUS).

**METHODS:**

It is a cross-sectional descriptive study based on secondary data from a historical series, referring to the purchase, volume, and the number of patients treated with different biological drugs (infliximabe, etanercept, adalimumab, rituximab, abatacept, tocilizumab, golimumab, and certolizumab pegol) for RA treatment in outpatient care from 2012 to 2017. The data were extracted from the SUS Outpatient Information System database-SIA/SUS and included ten drugs used for RA treatment. The study assessed the quantity and expenditure of these drugs, the number of RA patients treated, and the expenditure by RA subtypes. The National Broad Consumer Price Index was used to adjust the expenditures for December 2017.

**RESULTS:**

The Ministry of Health allocated approximately $500 million to provide about 2 million units of biological drugs for RA patients from 2012 to 2017. The supply of adalimumab 40 mg and etanercept 50 mg accounted for 68.3% of the total expenditure. The subtypes “other rheumatoid arthritis with rheumatoid factor” (ICD-10 M05.8), “rheumatoid arthritis without rheumatoid factor” (ICD-10 M06.0), and “Felty’s syndrome” (M05. 0) represented 84.5% of the total expenditures. The proportion of patients treated with biological drugs increased by 33.0%. There was a significant 83.0% increase in the number of patients using biological drugs compared to the overall number of RA patients treated during the study period.

**CONCLUSIONS:**

The results obtained allow us to draw a more recent profile of expenditure on RA treatment and indicate trends in the use of biological drugs for this condition, generating data that can support management decisions in public health policies.

## INTRODUCTION

Rheumatoid arthritis (RA) is a chronic, inflammatory, and disabling autoimmune disease characterized by peripheral synovitis and various extra-articular manifestations, primarily affecting women between the fourth and sixth decade of life^1^. It is a multifactorial and complex disease with an etiology that is still partially understood. It presents an estimated prevalence of 1% and exhibits highly variable average incidence across Brazil^2^. More frequent in women (3:1 women/men ratio), it can manifest at various levels of severity (disease activity), which is associated with patient survival and quality of life. RA significantly impacts economy^3^ since it can affect the ability to manage activities of daily life, including self-care, due to the joint damage and persistent pain. Specialists claim that tight control of inflammation is a desirable therapeutic strategy to decline disability rates in RA^4^.

The Brazilian Unified Health System (SUS) is known worldwide for providing universal assistance to citizens, including access to drugs. However, the commitment to provide this comprehensive assistance has increased public expenditure on health technologies, especially for chronic diseases. In this context, RA is a relevant disease attended by the pharmaceutical programs of the Ministry of Health (MoH) in the SUS, mainly due to the supply of biological drugs^5^.

Several compounds have been studied for the treatment of RA. The pharmacological treatment described in the Brazilian RA clinical guidelines published in 2017 by the MoH consists of (1) anti-inflammatory drugs; (2) immunosuppressors; (3) synthetic disease-modifying antirheumatic drugs (DMARDs); and (4) the following biological agents: adalimumab, certolizumab pegol, etanercept, infliximab, golimumab, abatacept, rituximab, and tocilizumab^1^. The first biological drug provided by SUS was infliximab in 2002. Later, in 2006, etanercept (25mg and 50mg) and adalimumab were included. The drugs golimumab, abatacept (IV formulation), rituximab, certolizumab pegol, and tocilizumab were provided by the public health system in 2013. Lastly, in 2015, abatacept subcutaneous was included, totalizing ten biological drugs^1,6^.

Studies that assess the health-related quality of life (HRQL) in patients with RA, before and after biological therapy treatment, have showed significant social, emotional, and physical improvements after the treatment^7,8^. A Brazilian study that evaluated the impact of biologic DMARDs on quality of life of rheumatic patients using the EuroQol five dimensions tool (EQ-5D) showed that patients with worse baseline degree of disability and quality of life (QoL) were the ones that presented the greater gain in QoL after 12 months of treatment. These findings reinforce the importance of biological drugs in the prognosis of RA^8^.

A retrospective database analysis to estimate total costs among patients with RA who persisted on or switched from newly initiated biologic drugs in the United States between from 2009 to 2014 showed that the costs per patient were $41,901 among persistent patients and $44,244 among switchers. The Etanercept appeared to be associated with the lowest costs^9^. In the USA, direct medical expenditure associated with drugs in RA patients was significantly higher (39.9%) compared to other medical costs (emergency, hospital inpatient, office-based visits)^10^.

In Brazil, from 2008 to 2010, the SUS allocated approximately one million US dollars to treat 103 patients with severe RA in Florianopolis (Santa Catarina State) , and the drugs accounted for 90.8% of the total expenditure^11^.

Drugs have presented financial impacts on the public health system, particularly biological drugs for the treatment of RA. There is a gap in the Brazilian public system regarding monitoring the use of health technologies after their incorporation. Therefore, expenditures studies become necessary to improve the information available for making informed decisions regarding technologies and facilitating analyses of funding sustainability.

This work aims to analyze data regarding quantity and expenditure of biological drugs used for RA treatment in outpatient public care in the SUS.

## METHODS

This cross-sectional descriptive study utilizes secondary data to examine the expenditure on biological drugs from the perspective of the governmental funding agency. It was possible to estimate the total expenditure of the Brazilian MoH considering the public disclosure of all government drug purchases in the Official Gazette of the Federal Republic of Brazil. Thus, unit prices for the investigated drugs were obtained for each year based on the available information. The amount that was exclusively supplied to RA patients each year was obtained (from SUS Outpatient Information System database- SIA/SUS) since some of these drugs are also purchased for other treatments. To present all data about the expenditure incurred over multiple years in US dollar currency, the methodology suggested by Turner et al.^12^ was applied, which is used to adjust the inflation and currency changes within health economic studies. The total expenditure by year in local currency (Brazilian Real) was inflated using local inflation rates - in this case, the National Broad Consumer Price Index (IPCA) for December 2017 was considered. After that, the values were converted to US$, considering the exchange rate to the same period (December 31st, 2017, 1 BRL = 0.302 USD)^13^.

Furthermore, a retrospective dataset from 2012 to 2017 was performed by collecting the SIA/SUS database data. These administrative records are compiled according to the production performed by subnational entities (states and municipalities) to serve as evidence for payment of services in SUS, which are open to public access^14^.

The data were analyzed and categorized using the free software Tabwin - developed by Brazilian MoH to disseminate public health data. These data were stratified by the annual amount of each biological drug dispensed and by the primary International Classification of Disease (ICD-10) codes listed in the Rheumatoid Arthritis clinical guidelines in Brazil^1^:

M05.0- Felty’s Syndrome, M05.1- Rheumatoid lung disease with rheumatoid arthritis factor, M05.2- Rheumatoid vasculitis with rheumatoid arthritis factor, M05.3- Rheumatoid heart disease with rheumatoid arthritis, M05.8- Other rheumatoid arthritis with rheumatoid factor, M06.0- Rheumatoid arthritis without rheumatoid factor, M06.8- Other specified Rheumatoid Arthritis and M08.0- Juvenile Rheumatoid Arthritis.

Thus, we were able to evaluate the quantities of dispensed drugs per year to measure the expenditure based on the unit price of each drug. Figure 1 illustrates the steps to extract information from the SIA-SUS database.

**Figure:**
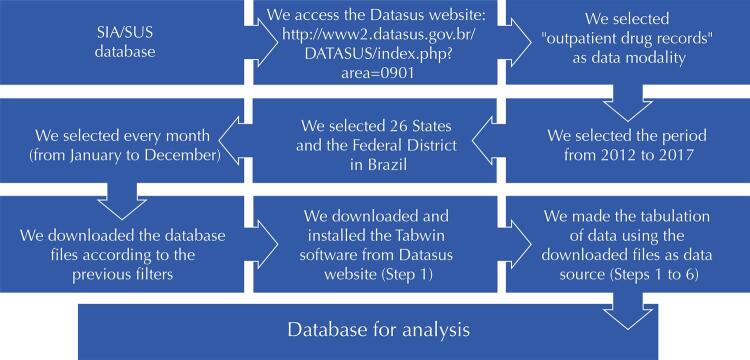
Steps to obtain outpatient data from SIA-SUS Database.

SIA/SUS: Outpatient Information System database – SIA/SUS

Since the SIA/SUS database accounts for number of services provided (procedures) instead of the number of distinct users, it was also necessary to associate all information to each user to evaluate the total number of users who consumed each drug. The same data extracted from SIA/SUS was systematized using the RStudio software, version R 3.5.1. Two premises were applied: (1) Capturing entries from individuals with the ICD-10 code of interest (M05.0, M05.1, M05.2, M05.3, M05.8, M06.0, M06.8, M08.0) and (2) Selecting the drug to be investigated (biological drugs used in RA treatment in SUS).

After eliminating duplicate cases (related to the same patient each year), the “distinct command” was applied to the patient’s unique identification variable - National Health Card (NHC), present in the SIA/SUS database. It is noteworthy that the NHC data is available in an encrypted form in this database, so personal and sensitive data that would allow patient identification are protected. Furthermore, by the analysis performed, it was not possible to identify treatment changes by the same patient due to the encryption previously mentioned. Thus, each unique combination of patient, ICD, and drug was considered an individual for this analysis. This last analysis showed the total amount of individuals who received each specific biological treatment per year and the total number of patients treated with biological or conventional synthetic drugs in outpatient public care for RA registered in the SIA/SUS system. From this step, the proportion between patients using biological drugs and the total number of RA patients treated over the years was assessed.

The characteristics of the records were analyzed by absolute and relative frequencies or average values. All the analyses were performed and stratified by each year’s data (2012–2017) and by the group of procedures.

This work used secondary and anonymized data from administrative and public databases of the Ministry of Health, which are open to the public. Thus, approval by the Research Ethics Committee was unnecessary.

## RESULTS

The study assessed the percentage participation of individuals in each treatment over the study period to demonstrate the magnitude to which new treatments were incorporated compared to the entire class of biologicals.

Five new biologicals drugs were incorporated and were fully available to the public system from 2013 to 2015: certolizumab pegol, abatacept 250 mg, rituximab 500 mg, golimumab 50 mg, and tocilizumab 20 mg/ml. The analysis showed that adalimumab 40 mg and etanercept 50 mg were the most used biological drugs among RA patients in SUS during all studied years (Table 1).

**Table 1:** Percentage of patients using biological drugs for RA, 2012-2017.

Drug	Year					

	2012	2013	2014	2015	2016	2017
Abatacept 125 mg S/C	-	-	-	-	0.05	1.56
Abatacept 250 mg IV	-	0.99	2.99	4.27	4.85	4.76
Adalimumab 40 mg S/C	46.50	45.53	39.72	37.19	34.44	32.27
Certolizumab Pegol 200 mg/ml S/C	-	0.13	1.25	2.25	3.78	4.77
Etanercept 25 mg IV	11.14	8.56	6.19	4.26	3.37	2.77
Etanercept 50 mg S/C	28.59	30.23	28.30	27.60	27.60	26.31
Golimumab 50 mg S/C	-	1.65	6.29	8.31	9.06	9.74
Infliximab 10 mg/ml IV	13.77	11.38	9.51	8.02	7.14	6.54
Rituximab 500 mg IV	-	0.54	2.16	2.92	3.50	4.04
Tocilizumab 20 mg/ml IV	-	0.99	3.59	5.18	6.21	7.24
Total	100	100	100	100	100	100

RA: rheumatoid arthritis; SC: subcutaneos; IV: intravenous; mg: miligrams; ml: milliliters.

From 2013, when other biological drugs were incorporated, a drop in the use of pre-existing treatments was evident, meaning that the replacement for new therapeutic options was usual, with emphasis on golimumab 50 mg and tocilizumab 20 mg/ml, which noticed the highest increase in the number of users. Conversely, adalimumab 40 mg showed the greatest decrease in medication usage (14.2%) during the same period (Table 1).

Additionally, by analyzing patient-centered data rather than procedure-centered data, it was possible to obtain data on the number of patients using biological drugs compared to the total number of outpatients treated for RA according to registered ICD-10 codes (Table 2).

**Table 2:** The relative number of RA patients treated under outpatient care in SUS, 2012–2017.

Year	Number of patients treated		% (a)/(b)

	Biological drug (a)	Total RA patients (b)	
2012	36,207	111,905	32.4
2013	41,098	121,803	33.7
2014	48,580	132,120	36.8
2015	54,787	139,294	39.3
2016	60,533	144,138	42.0
2017	66,244	153,737	43.1
Percentage increase 2012–2017	83.0	37.4	33.0

RA: rheumatoid arthritis.

This approach also showed that the assistance to RA patients by the SUS reached, in 2017, around 66,000 individuals using biological drugs. We observed a significant increase (37.4%) in drug treatment coverage for RA during this period. Expansion in the group of patients undergoing treatment with biological drugs was around 83% over the years, with 43.1% of all outpatients with RA treated in 2017. Besides, the higher increase occurred between 2013 and 2014 (18.2%), when the biological drugs incorporated in 2012 were effectively available to citizens (Table 2).

We also assessed the total quantity and expenditure of biological drugs supplied for RA treatment and over the years (2012–2017), along with the percentage contribution of each drug to the expenditure (Table 3). The expenditure reached about 500 million dollars in the acquisition of almost 2 million units of biological drugs during the evaluated period.

**Table 3:** Percentage contribution of each biological drug in expenditure, 2012-2017.

Drug	Quantity	Expenditure (USD)	Share in total expenditure (%)
Abatacept 125 mg	456,953	452,821.77	0.1
Abatacept 250 mg	7,137,412	9,553,089.09	1.9
Adalimumab 40 mg	105,483,888	253,469,185.73	51.2
Certolizumab Pegol 200 mg/ml	5,584,192	6,041,301.27	1.2
Etanercept 25 mg	2,462,304	8,491,202.32	1.7
Etanercept 50 mg	41,308,805	84,403,804.25	17.1
Golimumab 50 mg	45,357,672	55,018,755.19	11.1
Infliximab 10 mg/ml	23,959,791	57,484,619.04	11.6
Rituximab 500 mg	11,950,951	14,448,757.72	2.9
Tocilizumab 20 mg/ml	4,376,812	5,547,625.43	1.1
Total	1,976,177	494,911,161.80	100

Exchange rate: 1 BRL = 0,302 USD (December 31st, 2017)

RA: rheumatoid arthritis; SC: subcutaneos; IV: intravenous; mg: miligrams; ml: milliliters

Adalimumab 40 mg and etanercept 50 mg exhibited the biggest impact on the financial expenditure. They represented 68.3% of expenses with biological drugs, corroborating with the data shown in Table 1. Golimumab was the most significant financial expenditure among biological drugs incorporated after 2012 (Table 3).

The total quantity and expenditure of biological drugs supplied for RA treatment over the study period (2012–2017) are presented in Table 4. The average annual expenditure was 93 million dollars. From 2013 to 2015, expenditure increased at the same time that five new biological drugs were incorporated for RA treatment in the SUS. From 2015, annual expenditure presented a small decrease (-8.0%), which seems to be related to the increase in price competition after a more significant number of biological drugs became available. Stability was noted in federal expenditure on the purchase of biological drugs for RA treatment from 2012 to 2017. On the other hand, the quantity purchased (volume) grew by 97%.

**Table 4:** Quantity and expenditure (USD) of biological drugs for RA treatment by year, 2012-2017.

Drug	Year					

	2012	2013	2014	2015	2016	2017
					
	Expenditure	Expenditure	Expenditure	Expenditure	Expenditure	Expenditure
					
	(Quantity)	(Quantity)	(Quantity)	(Quantity)	(Quantity)	(Quantity)
Abatacept	0	280,884.67	2,185,988.24	2,200,963.90	2,419,586.68	2,465,665.60
250 mg		895	7,377	13,129	17,748	20,319
Abatacept	0	0	0	0	2,044.92	450,776.85
125 mg					20	4,111
Adalimumab	49,955,694.40	53,757,067.33	44,720,071.39	40,790,659.95	32,638,277.75	31,607,414.91
40 mg	110,689	126,068	135,793	143,712	149,716	154,107
Certolizumab	0	24,862.28	469,338.03	1,062,702.54	1,919,669.66	2,564,728.76
Pegol 200 mg/ml		89	2,542	6,228	12,653	18,210
Etanercept	2,481,392.33	2,130,897.74	1,457,401.76	1,068,749.56	736,225.46	616,535.47
25 mg	23,085	20,982	17,673	15,340	13,467	11,987
Etanercept	13,142,296.50	15,389,749.29	14,902,557.62	14,363,046.62	13,072,281.19	13,533,873.02
50 mg	61,133	75,768	90,357	103,078	114,825	121,701
Golimumab	0	1,213,520.88	8,553,601.75	13,542,637.97	15,185,999.18	16,522,995.41
50 mg		1,397	15,677	27,802	35,986	42,251
Infliximab	13,270,560.91	10,598,026.53	9,733,935.89	8,866,364.98	7,882,575.97	7,133,154.76
10 mg/ml	26,762	25,134	26,633	26,341	25,917	25,437
Rituximab	0	327,838.99	2,189,308.20	3,299,353.33	4,074,232.72	4,558,024.48
500 mg		388	2,930	4,727	6,460	8,153
Tocilizumab	0	120,178.57	1,122,171.29	1,149,641.44	1,472,623.15	1,683,010.98
20 mg/ml		904	8,940	16,824	23,850	30,862
Total	78,849,944.14	83,843,026.28	85,334,374.17	86,344,120.28	79,403,516.67	81,136,180.25
	221,669	251,625	307,922	357,181	400,642	437,138

Exchange rate: 1 BRL=0,302 USD (December 31^st^, 2017)

RA: rheumatoid arthritis; mg: miligrams; ml: millilite

By stratifying the data, it was also possible to assess the expenditure on biological drugs for RA treatment according to RA ICD-10 codes in public outpatient care in Brazil. The expenditure according to RA subtypes showed that M05.8- rheumatoid arthritis with rheumatoid factor was accounted for most of expenses (45.5%), followed by M06.0- rheumatoid arthritis without rheumatoid factor (22.5%), M05.0- ‘Felty’s syndrome (16.5), M06.8- Other specified Rheumatoid Arthritis (8.2%), M05.3- Rheumatoid heart disease with rheumatoid arthritis (4.6%), and M08.0- Juvenile Rheumatoid Arthritis (2.7%).

The ICD-10 M05.1 (Rheumatoid Lung Disease with rheumatoid arthritis) and M05.2 (Rheumatoid Vasculitis with rheumatoid arthritis) showed minimum participation in the percentage of expenses (0.19% and 0.06%, respectively).

## DISCUSSION

The biological drugs described in this study can be grouped into two classes of treatments according to their mechanism of action: anti-TNF (Tumoral Necrose Factor Inhibitors) or non-anti-TNF. The update of the guideline for RA therapy in 2015 recommended anti-TNF as the first choice due to their more accumulated evidence in use^15^. This criterion probably affected the diffusion speed of agents classified as non-anti-TNF. However, in the 2017 Brazilian guideline update, this recommendation was replaced for cost-minimization criteria (choosing the biological drug with the best cost/treatment ratio after the failure of synthetic DMARD agents)^1^. This recommendation is applied only to new patients or those with previous biological treatment failure. It means that patients who regularly use a biological drug with stable disease control should keep this therapy. Furthermore, it is essential to mention that some of these drugs are contraindicated for specific populations, such as subcutaneous abatacept, which is not recommended for children, thus it is not indicated for cases of juvenile rheumatoid arthritis^1^.

Administrative databases can be relevant for understanding disease care scenarios in public health systems such as the SUS. The data analysis in this study allowed us to understand how the cost of RA treatment based on biological drugs has evolved along with the expansion of RA outpatient pharmaceutical service in public health.

The growth in the expenditure and quantity of RA medicines over the study period is consistent with what was observed about the number of patients over the years. These findings are also consistent with the improved availability of treatments for RA patients over the years.

Studies have highlighted the increase in the availability of biological drug therapies for RA in the last decade, which led to reduction of absenteeism and presenteeism in the workplace of patients, allowing them to remain in the labor market for more years and reducing medical and indirect costs associated with the disease^16,17^.

Considering that MoH expenditure with biological medicines remained stable when comparing 2017 with 2012, we can see an increase in the quantity of supplied drugs and treated patients in the SUS, which may suggest the hypothesis of efficiency gains, but this requires further investigation.

This study also allowed us to know the drugs in charge of expressive expenditure by the MoH in the treatment of RA. According to data published about all drug sales in Brazil (public and private) by the Drug Market Regulation Chamber (CMED), there were revenues of about USD 4.6 billion in 2017, with the sale of more than 168 million units of biological drugs in Brazil^18^.

Adalimumab is placed second in the ranking of the 20 substances with the highest billings by pharmaceutical companies in 2017, with figures above USD 150 million. This ranking also includes infliximab (fourth position), rituximab (tenth position), and etanercept (eighteenth position), which are used both by the SUS and the private health care system for treatment of RA and other conditions such as ankylosing spondylitis, psoriatic arthritis, and psoriasis^18^.

The same report highlighted the increasing participation of the public sector in this market since the public purchases of biological drugs in this period corresponded to about 50% of the companies’ revenues in this sector, about USD 2.4 billion or 65.9 million units purchased^18^.

These data are consistent with our finding of an increase of biological drugs treatment in public care. The expenditure on biological drugs assessed in this study represented 2.06% of all Brazilian federal expenditure on drugs from 2012 to 2017, according to data published in 2019, showing that 4.38 billion dollars (exchange rate: 1 BRL=0,302 USD, December 31st, 2017) were spent by the federal government in this sector^19^. It means a significant expenditure, considering that an average of 75% of all drugs in SUS in that period was financed by the federal government^19^.

The highest expenditures according to RA subtypes were “other Rheumatoid Arthritis with rheumatoid factor (M05.8)” and “Rheumatoid arthritis without rheumatoid factor (M06.0).” Schneiders^20^ found the same pattern of expenditures.

Analysis based on secondary data about drug dispensing can also identify possible errors in hospital records. For instance, in the records related to Felty’s Syndrome (ICD M05.0), the data showed a significant decrease in the expenditure associated with this condition over the years. The same situation was reported by Silva et al.^6^, about some limitations of using administrative databases that may suffer from errors in recording the information and difficulty in determining Felty Syndrome, which can be confused with “pseudo-Felty Syndrome.” This is a rare condition among RA individuals worldwide, and it’s speculated that the inaccuracy of data is due to incorrect completion of the ICD-10 code in the administrative records^21^.

On the other hand, the M05.1 (Rheumatoid Lung Disease with rheumatoid arthritis) and M05.2 (Rheumatoid Vasculitis with rheumatoid arthritis) RA subtypes exhibited the lowest expenses with biological drugs, with no expense from 2014 onwards. This finding could be explained by the update of the RA clinical guidelines in 2013 that changed recommendations for treating these extra-articular manifestations, replacing biological drugs treatment for immunosuppressants^22^.

The scientific report that supported the incorporation of biological drugs for RA in the SUS had not pointed out significant differences between them in efficacy and safety for the main outcomes of the disease^23^. However, differences in the use among patients may be related to several factors, such as route of administration and dosage frequency (subcutaneous and weekly for etanercept; and intravenous and semiannual for rituximab, for example).

The drugs with the greatest increase in use were those with lower dosage frequency (once a month), such as golimumab (subcutaneous) and tocilizumab (intravenous). Considering that three oral targeted synthetic disease-modifying anti-rheumatic drugs (DMARDs) are the latest addition to the therapeutic options for rheumatoid arthritis in Brazil, future studies may identify significant changes in the trends of consumption established so far^24^.

An Argentine study listed the following attributes for choosing a biological drug in decreasing order of significance: cost, systemic adverse reactions, frequency of administration, efficacy, route of administration, local adverse events, and serious infections^25^.

Complementarily, a systematic review with 76 studies that aimed to assess the relationship between dosage and adherence to drug treatment pointed out that a longer interval between doses has a favorable impact on adherence^26^. Another study that assessed similar issues, specifically for RA, indicated the preference for treatments with fewer doses^27^.

About the route of administration, a study that investigated the preference for intravenous or subcutaneous administration concluded that health professionals and patients tolerate both routes^28^.

This study has some limitations. The absence of clinical data prevented us from assessing the real causes for the increase of patients treated with biological drugs, which is a limitation inherent to the data source. Since most of the current governmental databases are based on procedures rather than on users, it has been challenging to accurately evaluate the diffusion of new treatments, patients’ adherence, and impact of new drugs on the clinical outcomes. In addition, non-measurable errors can be incurred due to difficulties, inaccuracies, or omissions in feeding the databases.

The Brazilian MoH increasingly recognizes the need to monitor data after incorporating health treatments based on the patient’s journey. There is an imminent trend in the use of clinical data to support management decisions aimed at the financial sustainability of the public system. It is mandatory to advance the availability of comprehensive databases, which allow responses in the administrative and monitoring of clinical results.

## CONCLUDING REMARKS

In Brazil, there is a vast arsenal of information related to providing drug treatments and health procedures in the SUS. For many years, these databases have been used for administrative and financial purposes, but the need for post-incorporation analyses of health technologies, as well as monitoring the impact of new treatments and the profile of the users, especially in the context of high-cost drugs for chronic diseases, forces other destinations for them.

The data presented in this study allowed us to portray a scenario of recent years about the availability, federal expenditure, consumption, and dissemination of biological drugs for Rheumatoid Arthritis in Brazil. The analysis of administrative data also made it possible to find out which drugs contributed to this expense and which types of RA are most consumed, was along with the ratio of biological drugs from 2012 to 2017. Clinical studies with additional data would allow us to predict whether our findings are related to these drugs’ effectiveness or physician and user preferences. However, the results themselves reinforce the importance of studies with secondary data to support management decisions in public health policies.
